# Age and Sex-Dependent ADNP Regulation of Muscle Gene Expression Is Correlated with Motor Behavior: Possible Feedback Mechanism with PACAP

**DOI:** 10.3390/ijms21186715

**Published:** 2020-09-14

**Authors:** Oxana Kapitansky, Shlomo Sragovich, Iman Jaljuli, Adva Hadar, Eliezer Giladi, Illana Gozes

**Affiliations:** 1 The Elton Laboratory for Molecular Neuroendocrinology, Department of Human Molecular Genetics and Biochemistry, Sackler Faculty of Medicine, Sagol School of Neuroscience and Adams Super Center for Brain Studies, Tel Aviv University, Tel Aviv 6997801, Israel; oxana188@hotmail.com (O.K.); srshlomo@gmail.com (S.S.); advaad@gmail.com (A.H.); elieze@tauex.tau.ac.il (E.G.); 2 Department of Statistics and Operations Research, School of Mathematical Sciences, Raymond and Beverly Sackler Faculty of Exact Sciences, Tel Aviv University, Tel Aviv 6997801, Israel; jaljuli.iman@gmail.com; 3 Department of Molecular Genetics, Weizmann Institute of Science, Rehovot 7610001, Israel

**Keywords:** ADNP, NAP, muscle function, CatWalk, gene expression

## Abstract

The activity-dependent neuroprotective protein (ADNP), a double-edged sword, sex-dependently regulates multiple genes and was previously associated with the control of early muscle development and aging. Here we aimed to decipher the involvement of ADNP in versatile muscle gene expression patterns in correlation with motor function throughout life. Using quantitative RT-PCR we showed that *Adnp^+/−^* heterozygous deficiency in mice resulted in aberrant gastrocnemius (GC) muscle, tongue and bladder gene expression, which was corrected by the Adnp snippet, drug candidate, NAP (CP201). A significant sexual dichotomy was discovered, coupled to muscle and age-specific gene regulation. As such, Adnp was shown to regulate myosin light chain (*Myl*) in the gastrocnemius (GC) muscle, the language acquisition gene forkhead box protein P2 (*Foxp2*) in the tongue and the pituitary-adenylate cyclase activating polypeptide (PACAP) receptor PAC1 mRNA (*Adcyap1r1*) in the bladder, with PACAP linked to bladder function. A tight age regulation was observed, coupled to an extensive correlation to muscle function (gait analysis), placing ADNP as a muscle-regulating gene/protein.

## 1. Introduction

The activity-dependent neuroprotective protein (ADNP) [1,2], partly controlled by vasoactive intestinal peptide (VIP) and pituitary-adenylate cyclase activating polypeptide (PACAP) [3,4,5,6,7,8,9], is a known major regulator of gene function [10]. For example, our original studies showed that Adnp regulates more than 400 genes during embryonic development including genes controlling the development of the visceral endoderm, the heart and organogenesis in general [11]. In the adult mouse, Adnp regulates hundreds of genes important for brain and immune functions [12,13]. Importantly, there is a significant resemblance between mouse and human ADNP gene regulation [13,14,15]. The resemblance is further accentuated by the fact that mouse *Adnp* mRNA is 90% identical to human *ADNP* mRNA [2]. Indeed, the *Adnp*-deficient heterozygous (haploinsufficient) mouse model [16] predicted the autism-intellectual disability—associated ADNP syndrome [13].

Delayed motor development characterizes a large majority (96%) of *ADNP* syndrome children, suffering from de novo mutations in *ADNP* [14,17,18]. *ADNP* syndrome motor deficiencies manifest as hypotonia as well as opposing acute muscle tightness, signs of atrophy and abnormal gait [17,18]. The human pathological condition was mimicked by the mouse model of *Adnp* haploinsufficiency [13], exhibiting reduced muscle tone, as was demonstrated in the hanging wire and grip strength tests, along with gait deficits as established by the CatWalk gait analyses [13].

We have also shown that the expression levels of *ADNP* and its paralogue protein *ADNP2* in the vastus lateralis and bicep brachii muscles are significantly upregulated in the human elderly population, compared to young subjects, in a sex dependent manner. Thus, in the vastus lateralis *ADNP* is increased with aging in males and females, while *ADNP2* is increased with aging in females only. In the bicep brachii muscles, *ADNP* is increased with aging only in males and not in females, while *ADNP2* is increased with aging exclusively in females [19]. *ADNP* expression was highly correlated with 24 genes, with nicotinamide nucleotide adenylyl (NAD) transferase 1 (NMNAT1) being the leading gene/protein [19]. As such, NMNAT1-associated regulation of NAD^+^ salvage capacity in human skeletal muscle declines with aging, suggesting a causative or a compensatory role for ADNP content [20].

Importantly, not only the skeletal limb muscles are affected in the *ADNP* syndrome. For example, the bladder is slow in development with 81% of children suffering from the syndrome, exhibiting bladder training delay, and many are still not toilet trained when approaching puberty [17]. Another key characteristic of *ADNP* syndrome is speech delay, which presented in 98.6% of individuals and 19% had no language development at all [17]. Apraxia and absence of tongue movement was also found [18].

Mechanistically, ADNP is found in the nucleus as well as in the cytoplasm, and cytoplasmic representation increases in mature neurons, where ADNP is essential for neurite maintenance [21]. As such, ADNP exerts its control by regulating microtubule dynamics, binding to microtubule end binding proteins EB1 and EB3 [22], also regulating Tau-microtubule interaction [23,24] and axonal transport [12]. Importantly, cytoskeletal reorganization is associated with stretch-induced gene expression, implicating a role for cytoplasmic ADNP in gene expression regulation as well [25], especially in stretch-associated muscle cells.

*Adnp* deficiency is also associated with reduced autophagy that is dependent on microtubule integrity [26], with ADNP binding the microtubule associated protein 1 light chain 3B (LC3B), forming the autophagosome [27]. Interestingly, several muscle diseases present microtubule/autophagy deficits. For example, Duchenne muscular dystrophy (DMD) [28,29,30,31], Becker muscular dystrophy (BMD) [30,32,33], exhibiting absence or mutations in dystrophin, and tibial muscular dystrophy (TMD), exhibiting mutations in titin [34]. Furthermore, the ADNP regulating neuropeptide PACAP [3] was also shown to protect muscle function in a model of spinobulbar muscular atrophy (SBMA) [35].

The cytoplasmic interactions of ADNP with LC3 and EB1/EB3 are enhanced in the presence of the ADNP snippet, NAP (NAPVSIPQ, also known as CP201) containing an EB1/EB3 and self-interacting SxIP motif [12,23,24,26,27]. Moreover, through EB1/EB3 interactions, NAP enhances Tau-microtubule binding, protecting against tauopathy [23,24], which has also been found in the SOD1-G93A mouse model of the neuromuscular disorder, amyotrophic lateral sclerosis (ALS) [36].

Given the significant effect of ADNP mutations and ADNP deficiency on motor functions [13], and the strong association of ADNP with gene regulation (e.g., [10,11,21]), we hypothesized that it is involved in direct regulation of versatile muscle genes, with expression levels correlating with motor function throughout development. Our results have proven our hypothesis and identified multiple biomarkers for Adnp muscle activity.

## 2. Results

### 2.1. Adnp^+/–^ Mice Display Age-Dependent Aberrant Gene Expression in Skeletal Muscle, Tongue, and Bladder, Compared with Adnp^+/+^ Mice, Corrected by NAP Treatment

**Figure 1:**
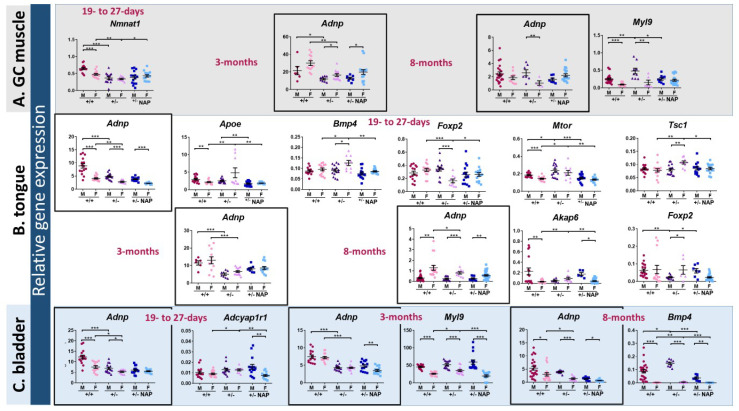
Activity-dependent neuroprotective protein (*Adnp*) haploinsufficiency alters muscle (GC muscle (**A**), tongue (**B**) and bladder (**C**)) gene expression in a sex- and age-dependent manner: NAP amelioration. Total RNA was extracted from 3 groups of age youngest group: 19–27-day-old mice (males: *Adnp^+/+^ n* = 5, *Adnp^+/–^ n* = 5, *Adnp^+/+^* NAP *n* = 4, *Adnp^+/–^* NAP *n* = 5; females: *Adnp^+/+^ n* = 5, *Adnp^+/–^ n* = 3, *Adnp^+/+^* NAP *n* = 4, *Adnp^+/–^* NAP *n* = 5). Young adults: 3-month-old mice (for muscle and tongue tissues males: *Adnp^+/+^ n* = 3, *Adnp^+/–^ n* = 4, *Adnp^+/+^* NAP *n* = 4, *Adnp^+/–^* NAP *n* = 4; females: *Adnp^+/+^ n* = 6, *Adnp^+/–^ n* = 7, *Adnp^+/+^* NAP *n* = 6, *Adnp^+/–^* NAP *n* = 8/9, respectively. For bladder of both sexes: *Adnp^+/+^ n* = 5, *Adnp^+/–^ n* = 5, *Adnp^+/+^* NAP *n* = 5, *Adnp^+/–^* NAP *n* = 5). Old group: 8-month-old mice (males: *Adnp^+/+^ n* = 8, *Adnp^+/–^ n* = 3, *Adnp^+/+^* NAP *n* = 4, *Adnp^+/–^* NAP *n* = 3; females: *Adnp^+/+^ n* = 6, *Adnp^+/–^ n* = 3, *Adnp^+/+^* NAP *n* = 5, *Adnp^+/–^* NAP *n* = 10/11 (in bladder and tongue *n* = 11)). Results were normalized to *Hprt*. The comparative Ct method was implemented here (2^−Δ*C*T^) for quantification of transcripts (indicated as relative gene expression and further explained in the Methods). A two-way ANOVA with Tukey’s post hoc test revealed significant differences between vehicle-treated *Adnp^+/+^* and *Adnp^+/–^* mice and between NAP and vehicle-treated *Adnp^+/–^* mice (* *p* < 0.05, ** *p* < 0.01 and *** *p* < 0.001). Sex differences were determined by an unpaired Student’s *t*-test.

To evaluate a potential mechanistic basis for the effects of ADNP on muscle development/maintenance, we examined the water-based DD- formulated/NAP-treated *Adnp^+/–^* and *Adnp^+/+^* mice, previously extensively studied for brain function and behavior (methods) [13]. We chose to assess gene expression patterns in skeletal muscle, tongue, and bladder as *ADNP* syndrome patients suffer from delays in motor development, language acquisition, and bladder training [17]. Given the developmental regulation attributed to Adnp as well as the sex-dependency [12], males and females at three age groups were chosen (methods): 19–27-days (youngest), 3-months (young adults), and 8-months (old group) (Figure 1). Based on previous gene arrays and RNA-seq experiments addressing Adnp gene regulation, 18 genes were chosen (Table S1). Using qRT-PCR, we measured transcript expression levels of these 18 genes including *Adnp* and the reference gene *Hprt* [37] in the youngest mouse group (19–27-day-old) in the gastrocnemius (GC) muscle (Figure 1A), tongue (Figure 1B), and bladder (Figure 1C). At 3- and 8-months of age, we selected the following genes, *Adnp, Adnp2, Akap6, Bmp4, Foxp2, Myl2* and *Myl9*.

We chose to concentrate on gene transcripts changed as a consequence of *Adnp* heterozygosity (haploinsufficiency), which were corrected by the ADNP snippet, drug candidate NAP, further attesting for specificity.

*Adnp* reduction as a consequence of *Adnp* gene copy deficiency (*Adnp^+/−^*) was significant in all tested tissues at the youngest tested age in both males and females, with significantly higher expression in *Adnp^+/+^* males compared with females (Figure 1A–C, boxes, the data for the GC muscle are not shown). This gene copy deficiency was maintained in the young adults (Figure 1A–C, boxes). However, it was abolished in the GC muscle of 8-month-old mice, while being maintained only in the female tongue and bladder (Figure 1A–C, boxes). Sex differences coupled to developmental and organ differences were observed in *Adnp* transcript content. For example, higher tongue *Adnp* expression was displayed in *Adnp^+/+^* males compared to other groups, in the youngest age tested, whereas, no sex differences were observed in the young adults and higher female expression was exhibited in the older group (Figure 1B, boxes). In contrast, increased male *Adnp* expression was observed in the bladder in selected genotype and treatment groups, in all tested ages, with all tested groups exhibiting this finding in the oldest tested age (Figure 1C, boxes). Of the 16 mRNAs assessed at the youngest age (Table S1 excluding *Adnp* and *Hprt*), *Myl2* (not shown, in preparation), *Myl9*, and *Nmnat1* showed Adnp/NAP as well as age/sex-dependent regulation in the GC muscle (Figure 1A).

Tongue sex/age/Adnp genotype/NAP-dependent regulation was shown in 5 out of the 16 tested genes at the youngest age tested (*Apoe, Bmp4, Foxp2, Tsc1* all in females and *Mtor*, in females and males) and two (*Akap6*, females and *Foxp2*, males) at the oldest age tested (Figure 1B), while only one transcript change per age group was shown in the bladder (Figure 1C, *Adcyap1r1*, young group females, *Myl9*, young adult females and *Bmp4*, old females and males).

As indicated above, we chose to concentrate on gene transcripts that have changed as a consequence of *Adnp* heterozygosity (haploinsufficiency), which were corrected by the ADNP snippet, drug candidate NAP (Figure 1). Additionally, Table S2 summarizes relative gene expression changes affected either by the *Adnp* genotype, or by NAP treatment and/or sex in muscle, tongue, and bladder in the three tested age groups. A most extensive sex difference was observed in the *Adnp^+/+^* mice indicating that the chosen studied gene transcripts present an inherent sexual difference, which was maintained, in part, in the *Adnp^+/−^* mice and also upon treatment with NAP. While most of the changes were subtle, there were a few exceptions as follows. *Akap6* increased by 3.71 folds in the 8-month-old *Adnp^+/−^* female GC muscle compared to *Adnp^+/+^* and *Myl2* decreased by 0.24 folds in the 3-month-old *Adnp^+/−^* vs. *Adnp^+/+^* male tongue, suggesting partial, but not complete NAP amelioration.

In conclusion, *Adnp^+/–^* mice displayed age-and sex-dependent aberrant gene expression in skeletal muscle, tongue, and bladder, compared with *Adnp^+/+^* mice and correction by NAP treatment. Of the selected tissues, the tongue showed the most robust changes, potentially connected with the *ADNP* syndrome phenotype of deficient language acquisition.

### 2.2. Muscle Aberrant Gene Expression Is Correlated with Adnp Deficiency in a Sex and Age-Dependent Manner

**Figure 2:**
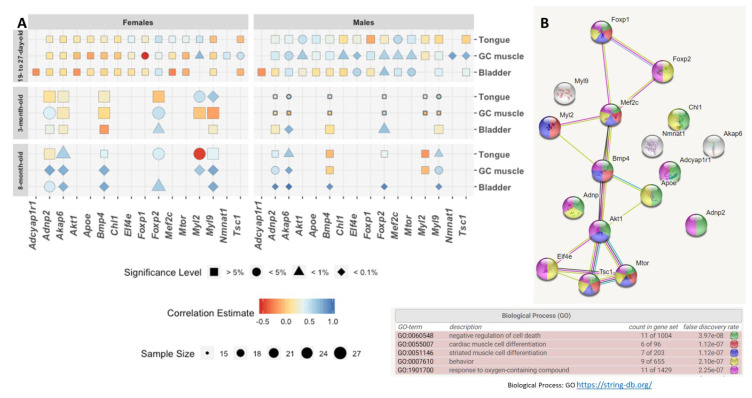
Heatmap correlations between *Adnp* expression levels and the expression levels of genes crucial for the proper function of GC muscle, tongue, and bladder. (**A**) Correlative analyses were performed using either the Pearson correlation coefficient method or the Spearman’s rank correlation coefficient, if at least one of the data sets was not normally distributed. Significant correlations were observed in a sex-dependent manner. The correlations were performed in the three tested age groups: 19–27-day-old, 3-month-old, and 8-month-old mice in three different tissues: GC muscle, tongue, and bladder. (**B**) Functional enrichment and network analysis of the gene protein products presented in panel A + Adnp. The genes/proteins presented a crucial network biological process role for proper muscle function as also previously revealed by mouse and human RNA-seq [12,14], as well as Affymetrix array [16] (Table S1).

Further comparisons of the expression levels of the 16 tested transcripts with *Adnp* expression revealed extensive correlations. *Akap6, Foxp2*, and *Myl9* showed the most abundant correlations across tissues, age, and sex (Figure 2A). Interestingly *Nmnat1*, which was correlated before with *ADNP* expression in human muscle [19], showed a highly significant correlation with Adnp in the GC muscle in the youngest group of males (Figure 2A). Comparing males to females suggested a more significant *Adnp* transcript correlation effect in males.

Developmentally, focusing on females, at the youngest age tested, almost no significant correlations were discovered, except for *Foxp1, Myl2,* and *Tsc1* in the GC muscle. This finding was in contrast to males, where 9 of 15 tested GC muscle gene transcripts showed significant correlation to *Adnp*, excluding *Foxp1* and *Myl2*. Again contrasting females, showing no *Adnp* gene transcript correlations at the youngest tested age in the bladder and the tongue, male results indicated *Akt1* and *Mef2c* correlations to *Adnp* in the tongue and of *Elf4e, Mtor,* and *Foxp2* in the bladder (Figure 2A). Additionally, *Nmnat1* correlated with *Adnp* in the GC muscle only in males and not in females. Finally, *Adcyap1r1*, encoding the PACAP-specific, PAC1 receptor, did not correlate with *Adnp* in the bladder (Figure 2A), despite the significant increase *Adcyap1r1* seen in the *Adnp^+/−^* versus *Adnp^+/+^* females and the correction of the female *Adnp^+/−^* levels to the *Adnp^+/+^* levels by NAP treatment (Figure 1C).

Surprisingly, at 3 months of age, no correlations were seen in the male GC muscle, while female *Adnp* correlated with *Adnp2* and *Foxp2. Foxp2* also correlated with *Adnp* in the bladder in 3-month-old males and females, whereas *Akap6* also correlated with *Adnp* only in males. Additionally, *Akap6* correlated with *Adnp* in the 3-month-old male tongue (but not female tongue), while *Myl9* correlated with *Adnp* in both sexes, and *Myl2* correlated with *Adnp* only in females (Figure 2A).

At 8 months of age, all gene transcripts tested showed significant correlations with *Adnp* in the bladder of males and females, with the highest correlations (almost 1) detected in males. Similarly, all transcripts tested showed significant correlations with *Adnp* in the female GC muscle, while only *Adnp2, Akap6,* and *Myl9* were significant in the males. *Akap6, Foxp2* and *Myl2* correlated with *Adnp* in the old female tongue, with *Akap6* also correlating with *Adnp* in males, and *Myl9* correlating with *Adnp* only in the male and not in the female tongue.

Interestingly, the most extensive (and only) negative correlation was observed in the females for *Foxp1* in the youngest GC muscle and for *Myl2* in the oldest tested tongue tissue (red, Figure 2A). Molecular interaction/STRING analysis showed the expected result indicating that a great majority of the tested transcripts protein products were linked to muscle development and differentiation (Figure 2B).

**Figure 3:**
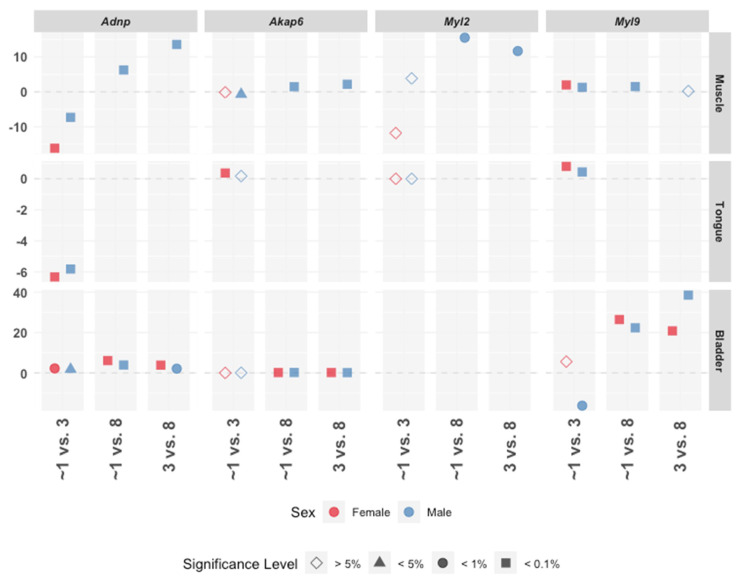
Age-dependent correlative gene expression patterns. Pairwise comparisons between age in month groups (x-axis), where the estimated difference (y-axis) is tested for statistical significance (shape) using post-hoc *t*-test with BH adjustment, for males and females separately (color). The most considerable differences were detected in *Adnp, Akap6, Myl2,* and *Myl9*. The majority of the comparisons were found statistically significant at 5% except for in 19–27-day-old mice = ~1 month vs. 3 months for *Akap6, Myl2,* and *Myl9* in muscle, tongue and bladder. In addition, 3- and 8-month-old males were found to be similar in the respective *Myl9* muscle expression levels.

Further analysis of GC muscle, tongue, and bladder gene expression across the three tested ages showed increased expression of *Adnp* and *Myl2* with age in the male muscle (blue), contrasting a decrease of *Adnp* in the female (pink), while a decrease in *Adnp* between one (i.e., 19–27 days) and three months of age was apparent in both sexes in the tongue (Figure 3). Age-dependent effects only on *Myl9* in the bladder were shown decreasing only in males at 3 months of age, compared to 1 month, and increasing thereafter in both males and females (Figure 3).

Together, the results here showed that muscle aberrant gene expression was correlated with *Adnp* deficiency in a sex, tissue, and age, development-dependent manner and associated key muscle regulating genes with Adnp function. The finding of the age, tissue, and female specific negative regulation of *Foxp1* and *Myl2*, emphasized age and sex-separation of the Adnp genotype.

### 2.3. Adnp^+/–^ Mice Display of Age-Dependent Aberrant Gene Expression in Skeletal Muscle is Correlated with Behavior

The youngest age examined (19–27-day-old) exhibited significant positive correlations with *Adnp* in 9 of the 15 tested genes (Figure 2) in the GC muscle of the male mouse. Hence, we further correlated specific RNA expression levels with previously published behavioral outcomes in the CatWalk gait measurements [13], (Table S3) addressing GC muscle function.

**Figure 4:**
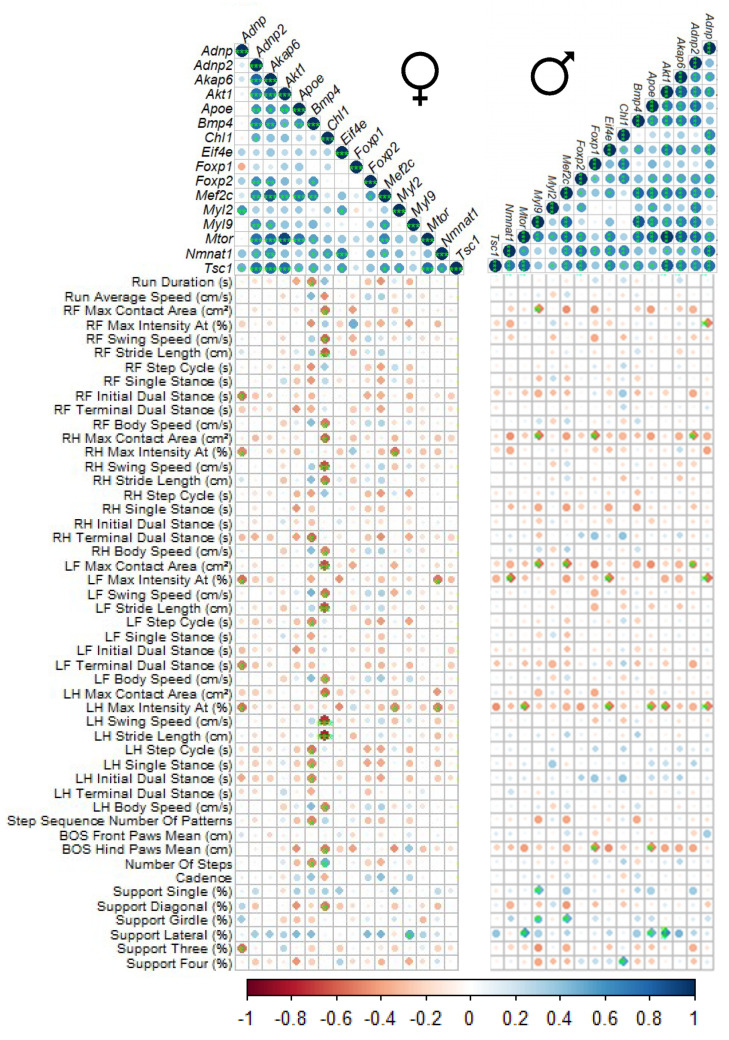
Correlative analysis between gene expression in GC muscle of 19–27-day old *Adnp* mice and the respective performance in the CatWalk apparatus. Correlative analyses were performed using Pearson correlation coefficient method. Positive correlations are displayed in a blue scale and negative correlations are displayed in a red-brown scale. The asterisks represent the significance level: * *p* < 0.05, ** *p* < 0.01 and *** *p* < 0.001. Please see Supplementary Table S3 for further explanation of the CatWalk measurements. R = right, L = left, H = hind paw, F = front paw.

As a word of introduction, the automated CatWalk gait analysis is an exceedingly sensitive tool allowing the identification of extensive number of gait and locomotion parameters with a minimal human interference [38]. This tool was previously implemented in the assessment of static and dynamic gait parameters in a variety of nerve injury models [39,40,41] including muscular dystrophies [39]. We show here numerous correlations between the different behavioral outcomes comparing males to females, with both sexes showing a similar, but not identical distribution patterns (Figure 4). Most tested gene transcripts showed correlations amongst each other and also with assorted CatWalk behaviors, displaying different patterns in males and females and implicating the involvement of these genes in muscle function. Interestingly, *Bmp4* and *Chl1* correlated with a number of different CatWalk behaviors in females (Figure 4, vertical view), while in the males, several genes correlated with selected CatWalk behaviors, e.g., maximal contact area (Figure 4, horizontal view).

Taken together, the *Adnp* genotype affected age-dependent gene expression in skeletal muscle in a sex-dependent correlation with gait parameters, with motor development/function being a major impediment in the *ADNP* syndrome patients.

## 3. Discussion

In the current paper we have discovered extensive correlation between *Adnp* expression and multi-muscle gene expression throughout life, with sex-specific patterns. *Adnp*-deficient gene regulation was corrected by its active site protein fragment, drug candidate NAP. Mechanistically, *Adnp* gene regulation was correlated with CatWalk motor behavioral outcomes.

Further referring to the mechanism, our original studies identified a high degree of correlation between *ADNP* and *NMNAT1* in the human muscle [19]. Here, a high correlation was discovered between *Adnp* and *Nmnat1* expression in the young male mouse muscle. NMNAT1 regulates NAD^+^ salvage capacity in human skeletal muscle, which is declining with aging [20].

Additionally, here, in the GC muscle, *Adnp* correlated with *Adnp2* only in the older mouse groups in a sex-dependent manner (appearing earlier and showing stronger correlations in the females). Previous findings linked dysregulation of *Adnp/Adnp2* correlations with aberrant synaptic function in neuropsychiatric diseases [42]. Interestingly, sexual differences were found in *ADNP, ADNP2* expression in Alzheimer’s disease (lymphocytes) [43] and in schizophrenia (postmortem brains and lymphocytes) [27,42]. While *ADNP2* is a less studied gene compared to *ADNP* [2,44,45], recent studies have also linked *ADNP2* deletion (together with other genes) to autism [46]. Further studies tied ADNP2 to osteoblast regulation [47], suggesting pleiotropic activities, similar, and potentially complementary to ADNP.

*Adcyap1r1* encoding the PACAP-specific, PAC1 receptor, did not correlate with *Adnp* in the bladder, despite the significant increase seen in the *Adnp^+/−^* females compared to *Adnp^+/+^* females and the amelioration with NAP treatment. These findings are of significant interest, as intra-bladder administration of the PAC1 receptor antagonist, PACAP(6–38), reduces urinary bladder frequency and pelvic sensitivity in mice exposed to repeated variate stress [48], with PACAP ameliorating *Adnp*-deficiency exacerbated stress response [3]. While ADNP was directly linked to cognitive impairment/language acquisition in humans [14,17,49] and in mice (vocalization) [13], indirect evidence also ties PACAP to the vocalization response [50]. We also participated in a study showing that PACAP regulates muscle function in protection against outcome measures in a mouse model of spinobulbar muscular atrophy (SBMA) [35].

Interestingly, here, we also found sexually/developmentally differential expression of *Myl2* and *Myl9* in correlation with *Adnp* expression. In this respect, MYL2 is linked to cardiac development and function [51], while MYL9 is involved in smooth muscle and non-muscle cell contractile activity e.g., in the gut and urinary tract [52], with *ADNP* syndrome children suffering cardiac as well as gastrointestinal problems [17]. Furthermore, a recent study identified Myl9 as highly important for skeletal muscle development [53] as well as to bladder and gastrointestinal smooth muscle contraction, with homozygous deletion leading to megacystis-microcolon-intestinal hypoperistalsis syndrome (MMIHS), a severe disease characterized by functional obstruction in the urinary and gastrointestinal tract [52,54]. Anatomically, the bladder divides into two parts: the dome and the base. The dome of the bladder is made up of smooth muscle, and the base consists of a trigone and neck that are closely connected to the pelvic floor. There are two urethral sphincters at the bladder outlet that are necessary for normal voluntary micturition [55]. Myl9 may be associated with the sphincters as well as the smooth muscle function. Together with the current findings, MYL9 may play a significant role in skeletal as well as smooth muscle regulation in the ADNP syndrome.

Similarly, Foxp2 is implicated as critical for central control for normal bladder voiding behavior [56] and may be important to tongue movement function [57], both affected in the *ADNP* syndrome. Indeed, *Foxp2* showed distinct sexual dichotomy in the tongue. Further sexual differences were observed in *Foxp2* also in correlation with Catwalk performance. Previous studies have indicated a close correlation of the *Foxp1/Foxp2* transcript expression and regulation of ultrasonic vocalization in mice. Foxp1 and the androgen receptor are co-expressed in striatal medium spiny neurons and brain-specific androgen receptor KO (Ar^NesCre^) mice exhibit reduced *Foxp1* expression in the striatum at E17.5 and P7.5 and an increased *Foxp2* level in the cortex at P7.5 [58]. Our previous bioinformatics results showed ADNP regulation of steroid pathways [15] and our current results suggest that this may be extended to the specific muscle cells. Furthermore, this steroid regulation may be linked with the higher prevalence of autism in boys compared to girls [58], with ADNP being a major autism driving gene [59,60]. Future research should further aim to decipher ADNP precise involvement in muscle function including cardiac and gastrointestinal muscles and use age and sex-dependency as major impacting study design and research outcome covariates.

While NAP treatment corrected many of the *Adnp^+/−^* associated deficiencies, it did not correct all (Table S2), as we have previously observed, also in other organs [13]. This could be due to additional differential temporal and sex-dependent controls exerted on these genes by Adnp and other regulatory proteins. One example is *Akap6* suggested as an important regulator of myoblast differentiation, myotube formation, and muscle regeneration [61]. Thus, further studies are required to elucidate the potential interactions between *Akap6* and *Adnp*.

## 4. Materials and Methods

### 4.1. Animals

The *Adnp^+/−^* mice, on a mixed C57BL and 129/Sv background, were previously described [13,16,59,62,63]. For continuous breeding, an ICR outbred mouse line was used [12,59]. Genotyping was performed by Transnetyx (Memphis, TN, USA). All animal groups were housed in a 12-h light/12-h dark cycle animal facility, with free access to rodent chow and water. All procedures involving animals were conducted under the supervision and approval of the IACUC of Tel Aviv University and the Israeli Ministry of Health (ethics certificate number: 01-18-018, valid dates: 15 March 2018–15 March 2021; ethics certificate number: 01-17-029: valid dates: 18 May 2018–6 April 2021). *Adnp*^+/+^ littermates served as controls.

### 4.2. Peptide Synthesis and Formulations

NAP peptide was custom synthesized using conventional methods [64], as before [13]. The peptide was dissolved in a vehicle solution termed DD, in which each milliliter included 7.5 mg of NaCl, 1.7 mg of citric acid monohydrate, 3 mg of disodium phosphate dihydrate, and 0.2 mg of benzalkonium chloride solution (50%) [13,65]. In days of scheduled behavioral tests, NAP was applied 2 h before the behavioral tests. For intranasal administration, each mouse was grasped in a vertical position and the solution was applied using a pipette and 5 μL tip. One droplet was released at the exterior naris and then inhaled by the mouse, and following a short break, the drug was administered into the other naris. This method has been extensively validated as producing reliable brain levels of NAP (CP201 also called davunetide) [66,67,68].

### 4.3. NAP Age Treatment Groups

Mice were divided to three age and treatment groups and sacrificed for RNA extraction by the end of the treatment period:

(1) A DD-group consisting of mouse pups that were subcutaneously administrated NAP (25 μg NAP/1 mL saline) for 21 consecutive days at the following doses: 40, 80, 120, and 160 μL on P9–P10, P11–P14, P15–P18, and P19–P21, respectively. At 21 days of age, these mice were treated daily with intranasal NAP for another 6 days [13] (tested at 19–27 days of age).

(2) 1-month-old mice were treated daily with intranasal NAP for 2 months (0.5 μg NAP/5 μL DD per mouse) (tested at 3 months of age) [13].

(3) 1-month-old mice (from group 1), were continuously treated daily with intranasal NAP for additional 7 months (0.5 μg NAP/5 μL DD per mouse) (tested at 8 months of age) [13].

### 4.4. Gait Analysis

CatWalk XT (Noldus Information Technology, Wageningen, the Netherlands) was used [13,69,70]. Importantly, parameters used are further described in Table S3.

### 4.5. RNA Extraction and cDNA Synthesis

Mice described above were sacrificed and the following tissues were immediately frozen with liquid nitrogen after excision: GC muscle, tongue, and bladder. Total RNA was isolated using TRI Reagent (T9424, Sigma-Aldrich, St. Louis, MO, USA) kit according to the manufacturer’s instructions, with slight modifications as described before [71]. Briefly, the tissues were added to tubes containing 1 mL of TRI Reagent and 5 × 2.8 mm stainless steel beads (D1033-28, Benchmark Scientific, Sayreville, NJ, USA). Subsequently, samples were homogenized for 10 cycles of 5 min using BeadBug microtube homogenizer (D1030-E, Daniel Biotech, Rehovot, Israel), subjected to centrifugation (12,000× *g*, 10 min, 4 °C) to pellet cell debris, and the supernatants were transferred to new tubes. All samples were processed as per the manufacturer’s protocol supplied with the TRI Reagent. The quantity and quality of RNA were analyzed by Nanodrop (ND-1000UV-Vis spectrophotometer, NanoDrop Technologies, Thermo Fisher Scientific, Waltham, MA, USA) [71]. Total RNA (1μg/sample) was subjected to reverse transcription with the qScript cDNA Synthesis Kit (Quanta Biosciences, Gaithersburg, MD, USA).

### 4.6. Quantitative Real-Time PCR

Real-time PCR was performed using PerfeCTa SYBR Green FastMix, Low ROX (Quanta Biosciences, Gaithersburg, MD, USA) and QuantStudio 12K Flex Real-Time PCR System (Thermo Fisher Scientific, Waltham, MA, USA), employing the default thermocycler program for all genes. RNA expression levels of each gene in each tissue were normalized to *Hprt* [37]. Primer pairs were designed using the IDT SciTools [72] and synthesized by Sigma (Sigma-Aldrich, St. Louis, MO, USA) (Table S1). Real-time PCR reactions were carried out in a total volume of 10μL in a 384-well plate (Applied Biosystems, Foster, CA, USA), containing Quanta PerfeCTa SYBR X2 and 300/350nM of each sense and antisense primers. All real-time PCR reactions were carried out in duplicates/triplicates. The relative expression of each gene in each sample was calculated with the 2^−Δ*C*T^ method [43,71], with Δ*C*T = *C*T (a target gene)−*C*T (the reference gene, i.e., *Hprt*) [73].

Biological and technical replicates were used for gene expression analysis as detailed in the Figure legends.

## 5. Conclusions

Despite the obvious study limitation of reliance upon subtle, albeit significant, gene expression changes, our results suggest a connection between *Adnp* and muscle regulation, during development and aging, including a potential involvement with the acto-myosin muscle system (*Myl2* and *Myl9*), energy metabolism (*Nmnat1*), and language acquisition *Foxp1/Foxp2* tongue expression. Our discovery of the extensive sexual dichotomy paves the path to better experimental design, looking separately at males and females both in basic research and most importantly in clinical studies. Our studies further suggest biomarkers for Adnp’s crucial activities regarding muscle function such as gait regulation and suggest a feedback mechanism with the ADNP regulator PACAP, with Adnp regulating bladder PAC1.

## Supplementary Materials

Supplementary materials can be found at https://www.mdpi.com/1422-0067/21/18/6715/s1.

## Abbreviations

ADNP	Activity-dependent neuroprotective protein
ALS	Amyotrophic lateral sclerosis
BMD	Becker muscular dystrophy
DMD	Duchenne muscular dystrophy
EB’s	Microtubule end-binding proteins
GC	Gastrocnemius
LC3B	Microtubule associated protein 1 light chain 3B
mRNA	Messenger ribonucleic acid
NAP	NAPVSIPQ
NMNAT1	Nicotinamide nucleotide adenylyl (NAD) transferase 1
PACAP	Pituitary adenylate cyclase activating polypeptide
PAC1	Pituitary adenylate cyclase-activating polypeptide type I receptor
qPCR	Quantitative real time PCR
SBMA	Spinobulbar muscular atrophy
VIP	Vasoactive intestinal peptide
