# Supplementary material for: Age and Sex-Dependent ADNP Regulation of Muscle Gene Expression Is Correlated with Motor Behavior: Possible Feedback Mechanism with PACAP
Source: Int J Mol Sci. 2020 Sep 14;21(18):6715. doi: 10.3390/ijms21186715 (PMC7555576; doi:10.3390/ijms21186715)
Supplement: Supplementary file 1 [file ijms-21-06715-s001.pdf]

Supplementary Tables:

Table S1. Mouse tested genes and specific primers utilized in qRT-PCR.

Gene Symbol and Name	Gene Description	Reason of Choice	Sense Primer (5'->3')	Antisense Primer (5'->3')
Adcyap1r1 *(Adenylate Cyclase Activating Polypeptide 1)*	This gene encodes type I adenylate cyclase activating polypeptide receptor, which is a membrane-associated protein and shares significant homology with members of the glucagon/secretin receptor family. This receptor mediates diverse biological actions of adenylate cyclase activating polypeptide 1 and is positively coupled to adenylate cyclase.	ADNP is regulated by vasoactive intestinal peptide (VIP), and pituitary adenylate cyclase-activating peptide (PACAP) [1]. In this respect, Pacap and its receptor PAC1 (Adcyap1r1) found to play a role in bladder pain syndrome (BPS)/interstitial cystitis (IC) [2].	AACCCGCTGCAAGACTTCTATGAC	TTAAGGATTTCGTGGGCGACA
Adnp *(Activity Dependent Neuroprotective Protein)*	This gene is an essential protein for brain formation and function [3, 4] and is crucial for normal cognitive performance [5]. Its activity is regulated by vasoactive intestinal peptide (VIP). Acts as a transcription factor regulating >400 genes during neurogenesis [4], controlling intracellular signaling cascades, angiogenesis [6] and heart development, neuronal migration and vital cellular functions. Mutation in this gene leading to ADNP syndrome (Helsmoortel-Van Der Aa Syndrome)/Adnp-Related Intellectual Disability within Autism Spectrum Disorder.	Studied gene- Found to be mutated in ADNP syndrome.	ACGAAAAATCAGGACTATCGG	GGACATTCCGGAAATGACTTT
Adnp2 *(Activity Dependent Neuroprotective Protein2)*	Adnp2 is a homologous protein of Adnp, providing cell protection. May be involved in transcriptional regulation. Since both Adnp and Adnp2 share 33% identity and 46% similarity, decreased amounts of Adnp will generally be compensated with Adnp2 higher levels as a compensatory effect [7, 8].	An important Adnp paralog protein. ADNP and ADNP2 precisely correlates in normal healthy conditions whereas in pathological conditions/cases (like schizophrenia and Alzheimer's disease) the correlation changes [7, 9, 10].	GGAAAGAAAGCGAGATACCG	TCCTGGTCAGCCTCATCTTC

Gene Symbol and Name	Gene Description	Reason of Choice	Sense Primer (5’->3’)	Antisense Primer (5’->3’)
Akap6 *(A-Kinase Anchoring Protein 6)*	The A-kinase anchor proteins (AKAPs) are a group of structurally diverse proteins, which have the common function of binding to the regulatory subunit of protein kinase A (PKA), confining the holoenzyme to discrete locations within the cell , and creating micro domains [11]. AKAPs organize PKA and its substrates into macromolecular complexes at specific subcellular locales [11]. Within the AKAPs, Akap6 is expressed in skeletal muscle among other places. Akap6 in collaboration with other AKAPs, anchor PKA to sites of excitation-contraction coupling, the sarcoplasmic reticulum, the NMJ, mitochondria and more [11]. Additionally, it was found that AKAP is important for skeletal myoblast differentiation and muscle regeneration [12].	Among the most downregulated genes found in the human RNA-seq comparing three ADNP-mutated LCLs to the healthy control line [13].	CGTCTCACAAAGCAGGACTGA	TCGTCCTCCACAGACACATC
Akt1 *(AKT Serine/Threonine Kinase 1)*	AKT1 is activated by platelet-derived growth factor. AKT1 Is a critical mediator of growth factor-induced neuronal survival, in the developing nervous system. Also it is a critical node in the signaling pathway: insulin like growth factor—phosphatidylinositol 3 kinase—Akt that has been implicated in muscle growth and regeneration after injury, in counteracting sarcopenia during aging, and in maintaining muscle cell viability [14].	AKT1 is an autism-related gene. AKT1 expression and phosphorylation are significantly reduced in autism spectrum disorder (ASD) brains [15, 16]. Furthermore, it is regulated by the *Adnp*$^{+/-}$ genotype and corrected by NAP (CP201) treatment in the mouse spleen [17].	CTTCTATGGTGCGGAGATTG	GAGGTTCTCCAGCTTCAGG
ApoE *(Apolipoprotein E)*	The protein encoded by this gene is a major apoprotein of the chylomicron. It binds to a specific liver and peripheral cell receptor and is essential for the normal catabolism of triglyceride-rich lipoprotein constituents. Also, it is a component of high density lipoproteins (HDL) and triglyceride-rich proteins (VLDL, chylomicrons), as such playing an important role in regulating lipid homeostasis [18]. Additionally, found in NMJs in mouse, rat and human skeletal muscle. At the NMJ at the NMJ its may originate from the motor neuron and be transported within axons [19].	Examining global gene expression profiles of *Adnp* KO mice vs. *Adnp* $^{+/+}$ and *Adnp* $^{+/-}$ mice at gestation day E9 using Affymetrix array revealed an upregulation in lipid transport genes in KO embryos (like Apoe) [4].	ACCGCTTCTGGGATTACCT	ATCAGTGCCGTCAGTTCTT
Bmp4 *(Bone Morphogenetic Protein 4)*	This gene encodes a secreted ligand of the TGF-beta (transforming growth factor-beta) superfamily of proteins. Ligands of this family bind various TGF-beta receptors leading to recruitment and activation of SMAD family transcription factors that regulate gene expression. The BMP pathway is a positive regulator of muscle mass	Performing RNAseq and gene array analysis of whole-mouse embryos, mouse brains and human ADNP-mutated lymphoblastoids significantly revealed dysregulation of bone/nervous system-	ATCACGAAGAACATCTGGAGAA	CTGCTGAGGTTGAAGAGGAA

Gene Symbol and Name	Gene Description	Reason of Choice	Sense Primer (5’->3’)	Antisense Primer (5’->3’)
	after disruption of the neuromuscular junction. Specifically, BMP4, are highly expressed in human fetal skeletal muscle side population and regulates myogenic progenitor proliferation [20, 21].	controlling genes resulting from ADNP mutation/deficiency (like Bmp4) [13].		
Chl1 *(Cell Adhesion Like) Molecule L1*	This protein is a close homolog of L1, and those proteins belong to a family of neural cell adhesion molecules. During development of the nervous system both proteins are widely expressed at relevant stages and participate in signal transduction pathways. Furthermore, these proteins are potent survival factors for motor neurons via the PI3K/Akt kinase and the MAP kinase pathways [22].	RNA sequencing (RNAseq) of human ADNP-mutated lymphoblastoids compared to a control cell line revealed an increase of >10 in the expression of CHL1 in ADNP-mutated lymphoblastoid cell lines. Interestingly, RNAseq analysis in mouse brains identified a significant downregulation in *Chl1* expression in *Adnp*$^{-/+}$ mice compared to littermate controls [13, 23]. Additionally, CHL1 is found to be involved in regulation of neuronal differentiation and survival, neurite outgrowth and axon guidance during development [24].	CACCGTGGATCAAAAATTC	CTGTTGAACGGAGAGTGGT
EIf4e *(Eukaryotic Translation Initiation Factor 4E)*	This protein is a component of the eukaryotic translation initiation factor 4F complex that aids in the translation initiation by recruiting ribosomes to the 5'-cap structure. Importantly, the availability of EIf4e is crucial for protein synthesis upon endurance training [25].	Autism-related gene [26]. Adnp directly binds to EIf4e protein, implicating Adnp as a potential regulator of protein translation in neuronal and glial cytoplasm [27]. Furthermore, hippocampal *eIF4E* expression was specifically increased in young *Adnp*$^{+/-}$ male mice compared to littermate control [27]. Also, a muscle function related gene [28].	TCTGGCTAGAGACACTGCTG	AGTCCATATTGCTATCTTATCACC
Foxp1 *(Forkhead Box P1)*	This gene, which belongs to subfamily P of the forkhead box (FOX) transcription factor family, is highly homologous to FOXP2, and can physically interact with FOXP2 (heterodimerize) to regulate transcription. It has roles in development, carcinogenesis, metabolism, and immunity. The protein contains both DNA binding	Autism and muscle related gene [30, 31].	GCGAGTAGAGAACGTTAAAGG	GGAAGGGTTACCACTGATCT

Gene Symbol and Name	Gene Description	Reason of Choice	Sense Primer (5’->3’)	Antisense Primer (5’->3’)
	and protein-protein binding domains. Mutation in this protein leading to a variety of pathologies including: language and speech defects, intellectual disability and/or autism spectrum disorder, facial dysmorphisms and motor deficits of the face and neck [29].			
Foxp2 *(Forkhead Box P2)*	This gene encodes a member of the forkhead/winged helix (FOX) family of transcription factors, which may bind directly to approximately 300 to 400 gene promoters in the human genome to regulate the expression of a variety of genes. The gene is required for proper development motor skills and spoken language. Also, has been considered as a potential susceptibility locus for the language deficits in autism or specific language impairments [32, 33].	Important for language acquisition regulated by ADNP and NAP (CP201) [34, 35].	TGGATTGAATGTATGTGTGG	CACGAAGACCTCAATGGTT
Hprt *(Hypoxanthine Phosphoribosyl-transferase 1)*	This gene encodes a transferase protein, which catalyzes conversion of hypoxanthine to inosine monophosphate and guanine to guanosine monophosphate via transfer of the 5-phosphoribosyl group from 5-phosphoribosyl 1-pyrophosphate. Also, it plays a central role in the generation of purine nucleotides through the purine salvage pathway.	Reference gene, highly suitable for studies on gastrocnemius muscle gene expression in comparison to other reference genes [36].	GGATTTGAATCACGTTTGTGTC	AACTTGCGCTCATCTTAGGC
Mef2c (Myocyte Enhancer Factor 2C)	This protein functions as a vital transcriptional regulator of skeletal muscle development, sarcomeric gene expression, fiber type control, and glucose uptake metabolism [37].	Regulates skeletal muscle development and function [38].	CGATGCAGACGATTCAGTAG	GTGGAACAGCACACAATCTTT
Myl2 *(Myosin Light Chain 2)*	Myl2 is an important protein involved in regulation of Myosin-ATPase activity and is activated by influx of calcium, which results in phosphorylation of Myl2 that triggers contraction. Defects in Myl2 can cause motor disabilities [39].	Gene expression profiling of *Adnp* KO mice compared with *Adnp* $^{+/+}$ and *Adnp* $^{+/-}$ mice at gestation day E9 revealed a cluster of genes downregulated in the KO embryos. One of the downregulated genes is *Myl2*, important for organogenesis [4]. Furthermore, Adnp binds Myl2 promoter.	GCCCTAGGACGAGTGAA	CCAAACATCGTGAGGAAC
Myl9 *(Myosin Light Chain 9)*	Myosin, a structural component of the muscle, consists of two heavy chains and four light chains. The protein encoded by this gene is a	RNA-seq analysis of ADNP mutated lymphoblastoid cells and a control	TGATAAGGAGGACCTGCAC	GCCCTCCAGATACTCGTCT

Gene Symbol and Name	Gene Description	Reason of Choice	Sense Primer (5’->3’)	Antisense Primer (5’->3’)
	myosin light chain that may regulate muscle contraction by modulating the ATPase activity of myosin heads. The encoded protein binds calcium and is activated by myosin light chain kinase.	lymphoblastoid cell line identified the most differentially expressed genes. One of the significantly downregulated genes is *MYL9* [40]. This results are consistent with mouse RNA-seq results showing decreased expression in hippocampus of *Adnp* $^{+/-}$ mice compared to littermate controls [23].		
Mtor *(Mechanistic Target of Rapamycin)*	The protein encoded by this gene belongs to a family of phosphatidylinositol kinase-related kinases. The MTOR signaling pathway combines both intracellular and extracellular signals and acts as a central regulator of cell metabolism, growth, proliferation and survival [41]. MTOR is an important regulator in maintaining skeletal muscle mass [42, 43].	Autism has been linked to alterations in PI3K/MTOR signaling pathway [44]. Furthermore, *Mtor* is regulated by *Adnp*$^{+/-}$ and corrected by NAP (CP201) in the mouse spleen [17].	GTACCGGCACACATTTGAAG	CGATCATCTCGATTCATACCC
Nmnat1 (Nicotinamide Nucleotide Adenylyltransferase 1)	The encoded enzyme is one of several nicotinamide nucleotides adenylyltransferases and is specifically localized to the cell nucleus. Activity of this protein leads to the activation of a nuclear deacetylase that functions in the protection of damaged neurons.	Correlative analysis between ADNP gene and 49 differentially expressed genes in young and old adults’ vastus lateralis identified *Nmnat1* the leading gene/protein [45].	GGTCGGTGATGCGTACAAGA	CCACGTATCCACTTCCACCC
Tsc1 (Tuberous Sclerosis 1)	This gene is a tumor suppressor gene that encodes the growth inhibitory protein hamartin. The encoded protein is involved in the signaling pathway cascade IGF1-PI3K-PKB/Akt-mTOR that controls protein synthesis and cell size [46].	Autism and vocalization related gene. Specifically, heterozygous or homozygous loss of *Tsc1* in mouse cerebellar Purkinje cell results in an autistic-like behaviors accompanied with vocalizations impediments [47].	CTCGAAGGTGGAAGACATTAG	AGCTGGTGTGACACAGAATAG

Table S2. Tabular summarization of significant fold-changes of the relative gene expression affected by ADNP genotype, NAP treatment and sex in: muscle, tongue and bladder at three age groups: A. 19-27-day-old mice B. 3-month-old mice C. 8-month-old mice.

Tissue	Age	Gene Symbol	Genotype Effect *Adnp*$^{+/-}$ vs. *Adnp*$^{+/+}$		NAP Treatment Effect *Adnp*$^{+/-}$ NAP vs. *Adnp*$^{+/-}$		Sex Effect Females vs. Males		
			Males	Females	Males	Females	*Adnp*$^{+/+}$	*Adnp*$^{+/-}$	*Adnp*$^{+/-}$ NAP
Gastrocnemius Muscle	19-27-day-old mice	*Adnp*	0.53	0.59			0.78		
		Adnp2					1.56		1.44
		Akap6							
		Akt1							
		Apoe				1.38			
		Bmp4					1.28		1.41
		Chl1	0.36					2.15	1.77
		Eif4e						0.82	
		Foxp1		1.36			0.78	1.30	1.41
		Foxp2							1.51
		Mef2c							1.38
		Myl2		0.50		1.74	1.85		
		Myl9					1.57	1.63	
		Mtor							1.50
		Nmnat1	0.57	0.72		1.23	0.73		
		Tsc1							
	3-month-old	*Adnp*	0.55	0.61				1.54	1.54
		Adnp2							
		Akap6					0.63		
		Bmp4							

		Foxp2							
		Myl2					2.23		2.23
		Myl9						1.42	
	**8-month-old**	*Adnp*						1.05	
		Adnp2	2.38						
		Akap6		3.71			0.56		2.29
		Bmp4					0.36	0.39	
		Myl2					2.08		1.96
		Myl9	1.90		0.51		0.37	0.31	
**Tongue**	**19-27-day-old mice**	*Adnp*	0.54	0.68			0.46	0.58	0.58
		Adnp2				2.21	0.65		
		Akap6			0.66		0.72	0.54	
		Akt1			0.61	0.70	0.71	0.80	
		Apoe		2.24	0.67	0.38	0.72		
		Bmp4		1.32		0.68		1.39	
		Chl1					0.69		
		Eif4e							
		Foxp1							
		Foxp2		0.49		1.61		0.47	
		Mef2c			0.57	0.69	0.81		
		Myl2							0.53
		Myl9					0.74		
		Mtor	1.27	1.47	0.64	0.63	0.71		
		Tsc1		1.38		0.78		1.34	
	**3-month-old**	*Adnp*	0.44	0.40					
		Adnp2		2.14	2.10			2.03	
		Akap6			4.23			2.68	0.44
		Bmp4							
		Foxp2		2.057				2.33	1.56
		Myl2	0.24					0.219	
		Myl9						0.59	0.99
		Adnp		0.63			4.15	3.19	2.11

	**8-month-old**	*Adnp2*						4.95	
		Akap6		0.67		0.46	0.60		0.24
		Bmp4		0.60			10.14	4.04	
		Foxp2	0.37		2.60			2.80	
		Myl9			2.42			0.87	
**Bladder**	**19-27-day-old mice**	*Adcyap1r1*		1.41		0.58			0.81
		Adnp	0.56	0.70			0.61	0.76	
		Adnp2		1.52			0.53		
		Akap6	0.68				0.61	0.67	0.64
		Akt1							
		Apoe					1.58		1.39
		Bmp4					0.53	0.70	
		Chl1		1.49			0.47	0.75	0.63
		Eif4e					0.78		0.77
		Foxp1					0.65		
		Foxp2						0.76	0.80
		Mef2c							
		Myl9	0.69				0.39	0.83	
		Mtor	0.75				0.71		
		Tsc1					0.68		
	**3-month-old**	*Adnp*	0.50	0.59					0.72
		Adnp2							0.68
		Akap6			1.63			1.25	
		Bmp4	1.31						
		Foxp2					0.80		0.60
		Myl9		1.35		0.56	0.57	0.67	0.33
	**8-month-old**	*Adnp*		0.45			0.56	0.34	0.47
		Adnp2			0.25		0.34	0.19	
		Akap6				0.39	0.14	0.31	
		Bmp4	1.68	2.67	0.25	0.18	0.02	0.03	0.02
		Foxp2				0.35	0.19	0.35	0.20
		Myl9				0.35	0.45		0.23

Table S3. Definitions of test parameters in CatWalk gait analysis.

**Run Characterization**	Run duration	The duration of the recorded run in sec.
	Cadence	Steps per second.
**Interlimb Coordination**	Swing speed	Speed (distance units/seconds) of a single paw during Swing (when not in contact with the glass plate).
	Body speed	Calculated by dividing the distance that the animal's body traveled from one initial contact of that paw to the next by the time to travel that distance.
	Step cycle	Is the time in seconds between two consecutive Initial Contacts of the same paw.
	BOS- front/ hind paws	The average width between either the front paws or the hind paws.
	Step Sequence	The Step Sequence lists the order in which the paws were placed on the glass plate.
	Support Three	This parameter displays the relative duration of simultaneous contact with the glass plate of all combinations of three paws.
	Support Diagonal	This parameter displays the relative duration of simultaneous contact with the glass plate of all combinations of diagonal paws.
	Support Lateral	This parameter displays the relative duration of simultaneous contact with the glass plate of all combinations of lateral paws.
**Temporal Parameters**	Single Stance	The duration in seconds of a single hind paw contact with a glass plate.
	Initial Dual Stance	The first time in a step cycle of a hind paw that the contralateral hind paw also makes contact with the glass plate.
	Terminal Dual Stance	The second step in a step cycle of a hind paw that the contralateral hind paw also makes contact with the glass plate.
	Max Intensity AT (%)	This is a ratio parameter, which reflects the relative intensity of both hind paws divided by the sum of the intensities of all four paws, i.e. Max Intensity At % $= \frac{RH+LH}{RF+LF+RH+LH}$*100
**Spatial Parameters:**	Mean Intensity of The 15 Most Intense Pixel	Referred to mean Intensity of the 15 pixels of a single paw with the highest intensity.
	Print Width	The width of an entire paw print in cm (vertical direction).

References:

1. Sragovich S, Ziv Y, Vaisvaser S, Shomron N, Hendler T, and Gozes I. The autism-mutated ADNP plays a key role in stress response. *Transl Psychiatry.* **2019**,9(1),235.
2. Ojala J, Tooke K, Hsiang H, Girard BM, May V, and Vizzard MA. PACAP/PAC1 Expression and Function in Micturition Pathways. *J Mol Neurosci.* **2019**,68(3),357.
3. Pinhasov A, Mandel S, Torchinsky A, Giladi E, Pittel Z, Goldsweig AM, Servoss SJ, Brenneman DE, and Gozes I. Activity-dependent neuroprotective protein: a novel gene essential for brain formation. *Brain Res Dev Brain Res.* **2003**,144(1),83.
4. Mandel S, Rechavi G, and Gozes I. Activity-dependent neuroprotective protein (ADNP) differentially interacts with chromatin to regulate genes essential for embryogenesis. *Dev Biol.* **2007**,303(2),814.
5. Vulih-Shultzman I, Pinhasov A, Mandel S, Grigoriadis N, Touloumi O, Pittel Z, and Gozes I. Activity-dependent neuroprotective protein snippet NAP reduces tau hyperphosphorylation and enhances learning in a novel transgenic mouse model. *J Pharmacol Exp Ther.* **2007**,323(2),438.
6. Dresner E, Malishkevich A, Arviv C, Leibman Barak S, Alon S, Ofir R, Gothilf Y, and Gozes I. Novel evolutionary-conserved role for the activity-dependent neuroprotective protein (ADNP) family that is important for erythropoiesis. *J Biol Chem.* **2012**,287(48),40173.
7. Malishkevich A, Marshall GA, Schultz AP, Sperling RA, Aharon-Peretz J, and Gozes I. Blood-Borne Activity-Dependent Neuroprotective Protein (ADNP) is Correlated with Premorbid Intelligence, Clinical Stage, and Alzheimer's Disease Biomarkers. *J Alzheimers Dis.* **2016**,50(1),249.
8. Zamostiano R, Pinhasov A, Gelber E, Steingart RA, Seroussi E, Giladi E, Bassan M, Wollman Y, Eyre HJ, Mulley JC, Brenneman DE, and Gozes I. Cloning and characterization of the human activity-dependent neuroprotective protein. *J Biol Chem.* **2001**,276(1),708.
9. Dresner E, Agam G, and Gozes I. Activity-dependent neuroprotective protein (ADNP) expression level is correlated with the expression of the sister protein ADNP2: deregulation in schizophrenia. *Eur Neuropsychopharmacol.* **2011**,21(5),355.
10. Merenlender-Wagner A, Malishkevich A, Shemer Z, Udawela M, Gibbons A, Scarr E, Dean B, Levine J, Agam G, and Gozes I. Autophagy has a key role in the pathophysiology of schizophrenia. *Mol Psychiatry.* **2015**,20(1),126.
11. Berdeaux R, and Stewart R. cAMP signaling in skeletal muscle adaptation: hypertrophy, metabolism, and regeneration. *Am J Physiol Endocrinol Metab.* **2012**,303(1),E1.
12. Lee SW, Won JY, Yang J, Lee J, Kim SY, Lee EJ, and Kim HS. AKAP6 inhibition impairs myoblast differentiation and muscle regeneration: Positive loop between AKAP6 and myogenin. *Sci Rep.* **2015**,5,16523.
13. Gozes I, Van Dijck A, Hacohen-Kleiman G, Grigg I, Karmon G, Giladi E, Eger M, Gabet Y, Pasmanik-Chor M, Cappuyns E, Elpeleg O, Kooy RF, and Bedrosian-Sermone S. Premature primary tooth eruption in cognitive/motor-delayed ADNP-mutated children. *Transl Psychiatry.* **2017**,7(2),e1043.
14. Rotwein P, and Wilson EM. Distinct actions of Akt1 and Akt2 in skeletal muscle differentiation. *J Cell Physiol.* **2009**,219(2),503.

15. Sheikh AM, Malik M, Wen G, Chauhan A, Chauhan V, Gong CX, Liu F, Brown WT, and Li X. BDNF-Akt-Bcl2 antiapoptotic signaling pathway is compromised in the brain of autistic subjects. *J Neurosci Res.* **2010**,88(12),2641.
16. Yasuda Y, Hashimoto R, Yamamori H, Ohi K, Fukumoto M, Umeda-Yano S, Mohri I, Ito A, Taniike M, and Takeda M. Gene expression analysis in lymphoblasts derived from patients with autism spectrum disorder. *Mol Autism.* **2011**,2(1),9.
17. Hacohen-Kleiman G, Sragovich S, Karmon G, Gao AYL, Grigg I, Pasmanik-Chor M, Le A, Korenkova V, McKinney RA, and Gozes I. Activity-dependent neuroprotective protein deficiency models synaptic and developmental phenotypes of autism-like syndrome. *J Clin Invest.* **2018**,128(11),4956.
18. Ali K, Lund-Katz S, Lawson J, Phillips MC, and Rader DJ. Structure-function properties of the apoE-dependent COX-2 pathway in vascular smooth muscle cells. *Atherosclerosis.* **2008**,196(1),201.
19. Akaaboune M, Villanova M, Festoff BW, Verdiere-Sahuque M, and Hantai D. Apolipoprotein E expression at neuromuscular junctions in mouse, rat and human skeletal muscle. *FEBS Lett.* **1994**,351(2),246.
20. Frank NY, Kho AT, Schatton T, Murphy GF, Molloy MJ, Zhan Q, Ramoni MF, Frank MH, Kohane IS, and Gussoni E. Regulation of myogenic progenitor proliferation in human fetal skeletal muscle by BMP4 and its antagonist Gremlin. *J Cell Biol.* **2006**,175(1),99.
21. Winbanks CE, Chen JL, Qian H, Liu Y, Bernardo BC, Beyer C, Watt KI, Thomson RE, Connor T, Turner BJ, McMullen JR, Larsson L, McGee SL, Harrison CA, and Gregorevic P. The bone morphogenetic protein axis is a positive regulator of skeletal muscle mass. *J Cell Biol.* **2013**,203(2),345.
22. Nishimune H, Bernreuther C, Carroll P, Chen S, Schachner M, and Henderson CE. Neural adhesion molecules L1 and CHL1 are survival factors for motoneurons. *J Neurosci Res.* **2005**,80(5),593.
23. Amram N, Hacohen-Kleiman G, Sragovich S, Malishkevich A, Katz J, Touloumi O, Lagoudaki R, Grigoriadis NC, Giladi E, Yeheskel A, Pasmanik-Chor M, Jouroukhin Y, and Gozes I. Sexual divergence in microtubule function: the novel intranasal microtubule targeting SKIP normalizes axonal transport and enhances memory. *Mol Psychiatry.* **2016**,21(10),1467.
24. Guseva D, Jakovcevski I, Irintchev A, Leshchyns'ka I, Sytnyk V, Ponimaskin E, and Schachner M. Cell Adhesion Molecule Close Homolog of L1 (CHL1) Guides the Regrowth of Regenerating Motor Axons and Regulates Synaptic Coverage of Motor Neurons. *Front Mol Neurosci.* **2018**,11,174.
25. Gautsch TA, Anthony JC, Kimball SR, Paul GL, Layman DK, and Jefferson LS. Availability of eIF4E regulates skeletal muscle protein synthesis during recovery from exercise. *Am J Physiol.* **1998**,274(2),C406.
26. Gkogkas CG, Khoutorsky A, Ran I, Rampakakis E, Nevarko T, Weatherill DB, Vasuta C, Yee S, Truitt M, Dallaire P, Major F, Lasko P, Ruggero D, Nader K, Lacaille JC, et al. Autism-related deficits via dysregulated eIF4E-dependent translational control. *Nature.* **2013**,493(7432),371.
27. Malishkevich A, Amram N, Hacohen-Kleiman G, Magen I, Giladi E, and Gozes I. Activity-dependent neuroprotective protein (ADNP) exhibits striking sexual dichotomy impacting on autistic and Alzheimer's pathologies. *Transl Psychiatry.* **2015**,5,e501.
28. Figueiredo VC, Englund DA, Vechetti IJ, Jr., Alimov A, Peterson CA, and McCarthy JJ. Phosphorylation of eukaryotic initiation factor 4E is dispensable for skeletal muscle hypertrophy. *Am J Physiol Cell Physiol.* **2019**,317(6),C1247.
29. Myers A, du Souich C, Yang CL, Borovik L, Mwenifumbo J, Rupps R, Study C, Lehman A, and Boerkoel CF. FOXP1 haploinsufficiency: Phenotypes beyond behavior and intellectual disability? *Am J Med Genet A.* **2017**,173(12),3172.

30. Araujo DJ, Anderson AG, Berto S, Runnels W, Harper M, Ammanuel S, Rieger MA, Huang HC, Rajkovich K, Loerwald KW, Dekker JD, Tucker HO, Dougherty JD, Gibson JR, and Konopka G. FoxP1 orchestration of ASD-relevant signaling pathways in the striatum. *Genes Dev.* **2015**,29(20),2081.
31. Frohlich H, Kollmeyer ML, Linz VC, Stuhlinger M, Groneberg D, Reigl A, Zizer E, Friebe A, Niesler B, and Rappold G. Gastrointestinal dysfunction in autism displayed by altered motility and achalasia in Foxp1 (+/-) mice. *Proc Natl Acad Sci U S A.* **2019**,116(44),22237.
32. Shu W, Cho JY, Jiang Y, Zhang M, Weisz D, Elder GA, Schmeidler J, De Gasperi R, Sosa MA, Rabidou D, Santucci AC, Perl D, Morrisey E, and Buxbaum JD. Altered ultrasonic vocalization in mice with a disruption in the Foxp2 gene. *Proc Natl Acad Sci U S A.* **2005**,102(27),9643.
33. Xu S, Liu P, Chen Y, Chen Y, Zhang W, Zhao H, Cao Y, Wang F, Jiang N, Lin S, Li B, Zhang Z, Wei Z, Fan Y, Jin Y, et al. Foxp2 regulates anatomical features that may be relevant for vocal behaviors and bipedal locomotion. *Proc Natl Acad Sci U S A.* **2018**,115(35),8799.
34. Vaisburd S, Shemer Z, Yeheskel A, Giladi E, and Gozes I. Risperidone and NAP protect cognition and normalize gene expression in a schizophrenia mouse model. *Sci Rep.* **2015**,5,16300.
35. Hacohen-Kleiman G, Yizhar-Barnea O, Touloumi O, Lagoudaki R, Avraham KB, Grigoriadis N, and Gozes I. Atypical Auditory Brainstem Response and Protein Expression Aberrations Related to ASD and Hearing Loss in the Adnp Haploinsufficient Mouse Brain. *Neurochem Res.* **2019**,44(6),1494.
36. Wang X, Zhao H, Ni J, Pan J, Hua H, and Wang Y. Identification of suitable reference genes for gene expression studies in rat skeletal muscle following sciatic nerve crush injury. *Mol Med Rep.* **2019**,19(5),4377.
37. Anderson CM, Hu J, Barnes RM, Heidt AB, Cornelissen I, and Black BL. Myocyte enhancer factor 2C function in skeletal muscle is required for normal growth and glucose metabolism in mice. *Skelet Muscle.* **2015**,5,7.
38. Potthoff MJ, Arnold MA, McAnally J, Richardson JA, Bassel-Duby R, and Olson EN. Regulation of skeletal muscle sarcomere integrity and postnatal muscle function by Mef2c. *Mol Cell Biol.* **2007**,27(23),8143.
39. Weterman MA, Barth PG, van Spaendonck-Zwarts KY, Aronica E, Poll-The BT, Brouwer OF, van Tintelen JP, Qahar Z, Bradley EJ, de Wissel M, Salviati L, Angelini C, van den Heuvel L, Thomasse YE, Backx AP, et al. Recessive MYL2 mutations cause infantile type I muscle fibre disease and cardiomyopathy. *Brain.* **2013**,136(Pt 1),282.
40. Grigg I, Ivashko-Pachima Y, Hait TA, Korenkova V, Touloumi O, Lagoudaki R, Van Dijck A, Marusic Z, Anicic M, Vukovic J, Kooy RF, Grigoriadis N, and Gozes I. Tauopathy in the young autistic brain: novel biomarker and therapeutic target. *Transl Psychiatry.* **2020**,10(1),228.
41. Laplante M, and Sabatini DM. mTOR signaling at a glance. *J Cell Sci.* **2009**,122(Pt 20),3589.
42. Yoon MS. mTOR as a Key Regulator in Maintaining Skeletal Muscle Mass. *Front Physiol.* **2017**,8(788.
43. Nader GA, McLoughlin TJ, and Esser KA. mTOR function in skeletal muscle hypertrophy: increased ribosomal RNA via cell cycle regulators. *Am J Physiol Cell Physiol.* **2005**,289(6),C1457.
44. Ganesan H, Balasubramanian V, Iyer M, Venugopal A, Subramaniam MD, Cho SG, and Vellingiri B. mTOR signalling pathway - A root cause for idiopathic autism? *BMB Rep.* **2019**,52(7),424.
45. Kapitansky O, and Gozes I. ADNP differentially interact with genes/proteins in correlation with aging: a novel marker for muscle aging. *Geroscience.* **2019**,41(3),321.

46. Bentzinger CF, Lin S, Romanino K, Castets P, Guridi M, Summermatter S, Handschin C, Tintignac LA, Hall MN, and Ruegg MA. Differential response of skeletal muscles to mTORC1 signaling during atrophy and hypertrophy. *Skelet Muscle.* **2013**,3(1),6.
47. Tsai PT, Hull C, Chu Y, Greene-Colozzi E, Sadowski AR, Leech JM, Steinberg J, Crawley JN, Regehr WG, and Sahin M. Autistic-like behaviour and cerebellar dysfunction in Purkinje cell Tsc1 mutant mice. *Nature.* **2012**,488(7413),647.
